# Time for Integrating Clinical, Lifestyle and Molecular Data to Predict Drug Responses

**DOI:** 10.1016/j.ebiom.2016.03.031

**Published:** 2016-03-25

**Authors:** Paola Patrignani, Melania Dovizio

**Affiliations:** Department of Neuroscience, Imaging and Clinical Sciences and CeSI-MeT, “G. d'Annunzio” University, School of Medicine, Chieti, Italy

The study performed by Pottegård and colleagues, published in this issue of *EBioMedicine* (Pottegård et al., 2016), falls within the clinically relevant scientific field which focuses on the variability in human drug response (Bruno et al., 2014, Fitzgerald, 2005). In some predisposed individuals, treated with a therapeutic dose of a drug, exaggerated responses (serious adverse events) can occur. These responses become evident when drugs, usually marketed at a limited number of doses, are broadly used in the population. Similarly, in some individuals drug therapies can result more effective than in the rest of exposed patients. The sources of heterogeneity among individuals in drug responses involve individual variability in genes, environment and lifestyle for each person. The occurrence of these determinants in drug response has provided the basis for the development of precision medicine, i.e., prevention and treatment strategies that take individual variability into account (FitzGerald, 2015, Collins and Varmus, 2015). While some advances in precision medicine have been made, the practice is not currently in use for most diseases.

Health-care information technology is advancing rapidly, creating new opportunities for the accurate reconstruction of comprehensive drug-exposure histories of individuals. Here, Pottegård et al. (2016) have used this approach to address their hypothesis that pharmaceutical agents may possess long-term carcinogenic or chemopreventive properties that are not identified by the premarketing genotoxicity and carcinogenicity testing or the preclinical trials. Thus, they performed a large-scale systematic screening for identifying associations between prescribed drugs and cancer risk using the high quality Danish nationwide health and demographic registries. Drugs were categorized according to the Anatomical Therapeutic Chemical (ATC) index, a hierarchical classification system developed by the WHO. Drugs are classified in groups at five different levels: first level, the organ or system on which they act; second and third levels indicate therapeutic/pharmacological subgroups; the fourth level is a therapeutic/pharmacological/chemical subgroup and the fifth level is the chemical substance.

The analyses consisted in two stages. In the first stage, they performed a conventional matched case–control approach using conditional logistic regression. In the second stage, those associations were examined according to two additional criteria: i) specificity, i.e., whether the drug was associated with a particular cancer or with cancer overall, and ii) dose–response relationship.

In this study, 22,125 drug-cancer pairs were evaluated. Among them, 1020 signals (i.e., drug-cancer associations) met the criteria for strength of association, specificity and dose–response pattern. The majority of the identified signals indicated an increase cancer risk and a smaller proportion of signals revealed a potential chemopreventive effect. The analysis of cancer risk association with the use of single drugs (fifth ATC level) (510 signals) suggested that 33% and 67% of them were linked to a reduction and an increase of cancer risk, respectively.

The screening algorithm was successful in identifying well-established causal associations, e.g., between female hormone and risk of breast cancer (International Agency for Research on Cancer, 2007). Moreover, it was confirmed an association between the photosensitizing antihypertensive drug hydrochlorothiazide and lip cancer (Friedman et al., 2012).

The use of the antiplatelet agent aspirin [acetylsalicylic acid, an inhibitor of thromboxane A_2_-dependent platelet activation (Patrignani and Patrono, 2015)] was inversely correlated with hepatocellular carcinoma (HCC) and larynx cancer. Interestingly, the use of the antiplatelet agent dipyridamole, an inhibitor of cAMP-phosphodiesterase which also exhibits anti-inflammatory and anti-oxidant properties (Weyrich et al., 2005), was inversely correlated with HCC. The protective effect of antiplatelet agents (such as aspirin and clopidogrel) has been demonstrated in a mouse model of hepatitis B virus-associated liver cancer (Sitia et al., 2012). Antiplatelet agents may act as anti-cancer drugs by inhibiting the release of several platelet mediators which may contribute to the development of chronic inflammation associated with HCC.

Several lines of evidence support the chemopreventive effect of the antiplatelet drug low-dose aspirin against colorectal cancer (Patrignani and Patrono, 2015) thus leading the United States Preventive Services Task Force to recommend its use for the primary prevention of cardiovascular disease and colorectal cancer (http://www.uspreventiveservicestaskforce.org/Page/Document/draft-recommendation-statement/aspirin-to-prevent-cardiovascular-disease-and-cancer). However, this protective signal was not detected in the present study.

Nonsteroidal anti-inflammatory drugs (NSAIDs) have been shown to have a chemopreventive effect in patients with sporadic colorectal adenomas via the inhibitory effect on the biosynthesis of the protumorigenic prostaglandin E_2_ (Patrignani and Patrono, 2015).This is confirmed in the present study showing that some individual drugs of the class, i.e., tolfenamic acid and diclofenac, were negatively associated with colon and rectum adenocarcinomas, respectively.

The study by Pottegård et al. (2016) has several points of strength including the detailed stratification according to cancer histology and clinical data collection in large-scale population which allowed evaluation of drug exposure for more rare cancers. However, it is characterized by important weakness related to the fact that this study lacks of adjustment for potential confounding from lifestyle, such as obesity, alcohol consumption, and smoking.

The approach of addressing hypotheses by the analysis of large-scale electronic medical records may capture unexpected responses to drugs in the population; however, the time is ripe to pursue new strategies that will take into account individual variability in genes, environment, and lifestyle for each person (Fitzgerald, 2005, 2015). The new approach integrates electronic medical records with biobank data to identify new disease pathways. Moreover, deep phenotyping of small numbers of patients will allow to elucidate the functional significance of genomic variation (Fitzgerald, 2005, 2015). This change in drug development will permit the use of safer and more efficacious medicines.

## Disclosure

PP receives grant from Associazione Italiana Ricerca sul Cancro, Ministero dell'Istruzione, dell'Università e della Ricerca (MIUR), and personal fees from Bayer. MD declared no conflicts of interest.
